# *N*-methyl-D-aspartate receptors mediate activity-dependent down-regulation of potassium channel genes during the expression of homeostatic intrinsic plasticity

**DOI:** 10.1186/s13041-015-0094-1

**Published:** 2015-01-20

**Authors:** Kwan Young Lee, Sara E Royston, Max O Vest, Daniel J Ley, Seungbae Lee, Eric C Bolton, Hee Jung Chung

**Affiliations:** Department of Molecular and Integrative Physiology, University of Illinois at Urbana-Champaign, 407 South Goodwin Avenue, 524 Burrill Hall, Urbana, IL 61801 USA; Program in Neuroscience Program, University of Illinois at Urbana-Champaign, Urbana, Illinois 61801 USA; Medical Scholars Program, University of Illinois at Urbana-Champaign, Urbana, Illinois 61801 USA

**Keywords:** Homeostatic intrinsic plasticity, Potassium channel, NMDA receptor, Action potential, Hippocampus

## Abstract

**Background:**

Homeostatic intrinsic plasticity encompasses the mechanisms by which neurons stabilize their excitability in response to prolonged and destabilizing changes in global activity. However, the milieu of molecular players responsible for these regulatory mechanisms is largely unknown.

**Results:**

Using whole-cell patch clamp recording and unbiased gene expression profiling in rat dissociated hippocampal neurons cultured at high density, we demonstrate here that chronic activity blockade induced by the sodium channel blocker tetrodotoxin leads to a homeostatic increase in action potential firing and down-regulation of potassium channel genes. In addition, chronic activity blockade reduces total potassium current, as well as protein expression and current of voltage-gated K_v_1 and K_v_7 potassium channels, which are critical regulators of action potential firing. Importantly, inhibition of *N*-Methyl-D-Aspartate receptors alone mimics the effects of tetrodotoxin, including the elevation in firing frequency and reduction of potassium channel gene expression and current driven by activity blockade, whereas inhibition of L-type voltage-gated calcium channels has no effect.

**Conclusions:**

Collectively, our data suggest that homeostatic intrinsic plasticity induced by chronic activity blockade is accomplished in part by decreased calcium influx through *N*-Methyl-D-Aspartate receptors and subsequent transcriptional down-regulation of potassium channel genes.

**Electronic supplementary material:**

The online version of this article (doi:10.1186/s13041-015-0094-1) contains supplementary material, which is available to authorized users.

## Background

Chronic perturbations in neuronal activity trigger homeostatic intrinsic plasticity, which maintain neuronal excitability within physiologic boundaries [1]. For example, action potential (AP) firing frequency is elevated by chronic activity blockade in cultured hippocampal and cortical neurons [2-6] and in hippocampus *in vivo* [7]. Conversely, chronic activity enhancement leads to a reduction in AP firing rate [2,5,8]. The elevation in AP firing frequency induced by global activity suppression is coupled to elevated sodium (Na^+^) current density and reduced potassium (K^+^) current density in dissociated cortical neurons [4], implicating activity-dependent changes in ionic conductance in homeostatic intrinsic plasticity. Intrinsic firing properties of mammalian neurons are largely determined by the biophysical properties, spatial distribution, and abundance of ion channels at the plasma membrane [9]. However, the identity of the specific channels critical for homeostatic intrinsic plasticity remains largely unknown.

Recent studies have reported that long-term changes in intracellular calcium (Ca^2+^) concentration can regulate expression of multiple ion channels [10] and mediate homeostatic plasticity in response to chronic alterations in neuronal activity [2,11-15]. In particular, prolonged inhibition of Ca^2+^ influx through *N*-Methyl-D-aspartate receptors (NMDAR) but not L-type voltage-gated Ca^2+^ channels (VGCC) has been shown to mimic the elevation in firing frequency driven by chronic activity blockade [2]. Interestingly, inhibition of transcription blocks induction of homeostatic plasticity at excitatory synapses in cultured cortical and hippocampal neurons [11,14], suggesting a critical role for transcriptional regulation of gene expression in homeostatic plasticity. Furthermore, the computational modeling work has recently shown that activity-dependent regulation of ion channel transcripts can underlie a mechanism by which alterations in intracellular Ca^2+^ concentration can control neuronal homeostasis [16]. Since Ca^2+^ influx through NMDARs or L-type VGCCs stimulates activity-dependent transcription in neurons [17], we hypothesized that homeostatic intrinsic plasticity is mediated in part by activity-dependent expression of genes whose protein products regulate intrinsic properties of neurons.

In the present study, we used unbiased gene expression profiling, real-time quantitative polymerase chain reaction (QPCR), and electrophysiology to search for novel molecular players critical for modulating ionic currents during the induction of homeostatic intrinsic plasticity in high-density cultures of dissociated hippocampal neurons. We identified 873 activity-dependent transcripts whose protein products have not previously been implicated in homeostatic plasticity. We found that chronic activity blockade leads to an increase in firing frequency, and decreases in K^+^ current and gene expression of various K^+^ channels. Furthermore, we discovered that the observed homeostatic intrinsic plasticity and down-regulation of K^+^ channel genes are mediated by changes in NMDAR activity but not L-type VGCC activity.

## Results and discussion

### Chronic activity blockade induces a homeostatic increase in intrinsic excitability

**Figure 1:**
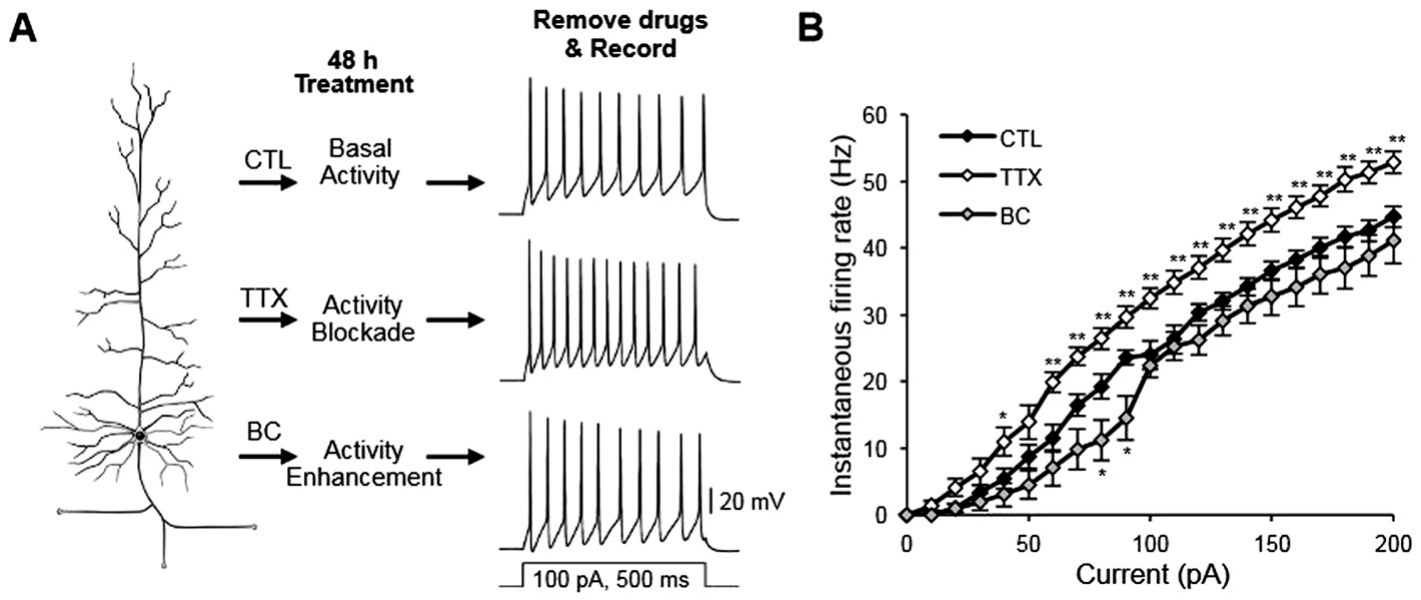
**Chronic blockade of neuronal activity induces a homeostatic increase in intrinsic excitability. (A,B)** Whole-cell patch clamp recording of rat dissociated hippocampal neurons cultured in a high density (DIV 12–14) that were treated for 48 h with vehicle control (CTL-H_2_O, 0.1% H_2_O), TTX (0.5-1 μM), or BC (20 μM). Following treatment removal, spike trains were evoked in pyramidal neurons by delivering constant somatic current pulses for 500 ms duration at a resting potential of −60 mV. **(A)** Representative spike trains. **(B)** Average AP firing rates (Hz) measured in pyramidal neurons treated for 48 h with CTL-H_2_O (n = 13), TTX (n = 10), or BC (n = 9). TTX but not BC treatment for 48 h significantly increased AP firing frequency compared to CTL-H_2_O treatment. Mean ± SEM (*p < 0.05, **p < 0.01).

To collect ample amounts of RNA for cDNA microarray analysis, high-density cultures of dissociated hippocampal neurons (330 neurons/cm^2^) were treated for 48 hours (h) with vehicle control (CTL-H_2_O, 0.1% H_2_O), the sodium channel blocker tetrodotoxin (TTX, 0.5-1 μM), or the GABA_A_ receptor antagonist bicuculine (BC, 20 μM) (Figure 1A). Application of TTX immediately blocked spontaneous activity of hippocampal neurons whereas BC application rapidly induced burst firing of action potentials compared to CTL-H_2_O (Additional file 1: Figure S1). To verify the induction of homeostatic intrinsic plasticity, AP firing frequency was measured immediately after TTX or BC was washed out from the neurons treated for 48 h with TTX or BC as previously described [2] (Figure 1A,B).

TTX treatment for 48 h significantly increased AP firing frequency compared to CTL treatment at 40 pA injection (*p* < 0.05) and for all current injections over 60 pA in hippocampal neurons cultured at high density (Figure 1A,B, *p* < 0.01). The mean AP firing frequency in the TTX-treated neurons induced by 100 pA current injection was 32.4 ± 1.5 Hz, a 35% increase compared to CTL-H_2_O neurons (23.9 ± 2.1 Hz, *p* < 0.01). In contrast, 48 h BC treatment significantly reduced AP firing rates at 80–90 pA injections but did not alter firing rates for all other current injections (Figure 1A,B). Although membrane capacitance and resting membrane potential were unaffected by TTX or BC treatment (Table 1), TTX treatment increased the input resistance (Table 1) and decreased fast after-hyperpolarization (fAHP) amplitude and AP latency compared to CTL-H_2_O treatment (Table 2). Taken together, these findings indicate that TTX treatment for 48 h but not BC treatment caused an increase in AP firing rates in hippocampal neurons cultured at high density.

**Table 1:** **Passive properties of hippocampal pyramidal neurons cultured at high density following 48 h pharmacological treatment**

**Treatment**	***n***	***C***_m_ **(pF)**	***R***_in_ **(MΩ)**	***V***_m_ **(mV)**
CTL-H_2_O	31	48.8 ± 1.2	571 ± 34	−59.5 ± 0.6
TTX	20	47.1 ± 1.9	721 ± 44*	−59.8 ± 0.9
BC	9	48.0 ± 3.4	529 ± 53	−60.1 ± 1.7
APV	9	45.0 ± 1.8	787 ± 51*^,#^	−58.0 ± 2.1
TTX + APV	10	50.7 ± 2.4	791 ± 22**^,#^	−58.7 ± 0.8
TTX + Nif	9	47.4 ± 3.1	769 ± 22*^,#^	−58.1 ± 0.6
CTL-DMSO	12	49.3 ± 1.5	583 ± 32	−60.5 ± 0.9
Nif	9	46.8 ± 2.3	672 ± 43	−58.7 ± 0.5
STO-609	10	50.2 ± 2.0	680 ± 63	−60.7 ± 0.8

*n*, number; *C*m, Whole-cell membrane capacitance; *R*in, input resistance; *V*m, resting membrane potential. Each value represents the mean ± SEM (*p < 0.05, **p < 0.01 for CTL-H_2_O vs. drug treatment; ^#^ *p* < 0.05 for BC vs. other drug treatment).

**Table 2:** **AP properties of hippocampal pyramidal neurons cultured at high density following 48 h pharmacological treatment**

**Treatment**	***V***_T_ **(mV)**	**AP height (mV)**	**AP rise (ms)**	**AP decay (ms)**	**AP HW (ms)**	**fAHP (mV)**	**AP latency (ms)**
CTL-H_2_O	−39.4 ± 0.5	64.6 ± 1.3	0.70 ± 0.03	1.72 ± 0.07	1.94 ± 0.07	−19.1 ± 0.6	15.1 ± 0.72
TTX	−40.2 ± 0.7	67.4 ± 1.5	0.67 ± 0.03	1.81 ± 0.08	1.97 ± 0.10	−15.4 ± 0.7**	11.2 ± 0.58**
BC	−37.6 ± 0.4	65.4 ± 2.5	0.60 ± 0.03	1.48 ± 0.14	1.55 ± 0.10	−19.7 ± 1.4^#^	14.5 ± 0.87
APV	−38.7 ± 1.5	65.6 ± 3.2	0.89 ± 0.05*^,##,^^^	2.52 ± 0.17**^,##,^^^	2.56 ± 0.07**^,##,^^^	−11.5 ± 1.1**^,^^^	12.9 ± 1.12
TTX + APV	−39.9 ± 0.7	62.8 ± 2.2	0.70 ± 0.02	1.92 ± 0.12^$^	2.18 ± 0.12^^^	−15.8 ± 0.9	12.3 ± 0.78
TTX + Nif	−38.4 ± 0.7	63.5 ± 2.3	0.62 ± 0.05^$$^	1.78 ± 0.13^$$^	2.04 ± 0.16	−17.6 ± 1.2^$$^	11.0 ± 0.54*
CTL-DMSO	−39.0 ± 0.9	66.5 ± 1.4	0.76 ± 0.07	1.73 ± 0.19	2.04 ± 0.11	−17.9 ± 1.5	16.1 ± 1.5
Nif	−38.0 ± 0.7	68.2 ± 3.2	0.69 ± 0.05	1.92 ± 0.13	2.07 ± 0.12	−17.5 ± 0.8	16.9 ± 1.0
STO-609	−40.0 ± 0.9	69.3 ± 1.4	0.78 ± 0.05	2.14 ± 0.12	2.26 ± 0.13	−15.9 ± 0.9	16.8 ± 2.0

*V*T, voltage threshold for action potential; AP, Action potential; rise, 10-90% rise time of AP; decay, 10-90% decay time of AP; HW, half-width; fAHP, fast after-hyperpolarization. AP properties were measured from the first action potential evoked by a current step to 100 pA at a holding potential of −60 mV. Each value represents the mean ± SEM (*p < 0.05, **p < 0.01 for CTL-H_2_O vs. drug treatment; ^#^ *p* < 0.05, ^##^ *p* < 0.01 for TTX vs. other drug treatment; ^^^ *p* < 0.05, ^^^^ *p* < 0.01 for BC vs. other drug treatment; ^$^ *p* < 0.05, ^$$^ *p* < 0.01 for APV vs. other drug treatment).

### Microarray analysis reveals that chronic alteration of network activity regulates genes involved in hippocampal synaptic and intrinsic membrane properties

**Figure 2:**
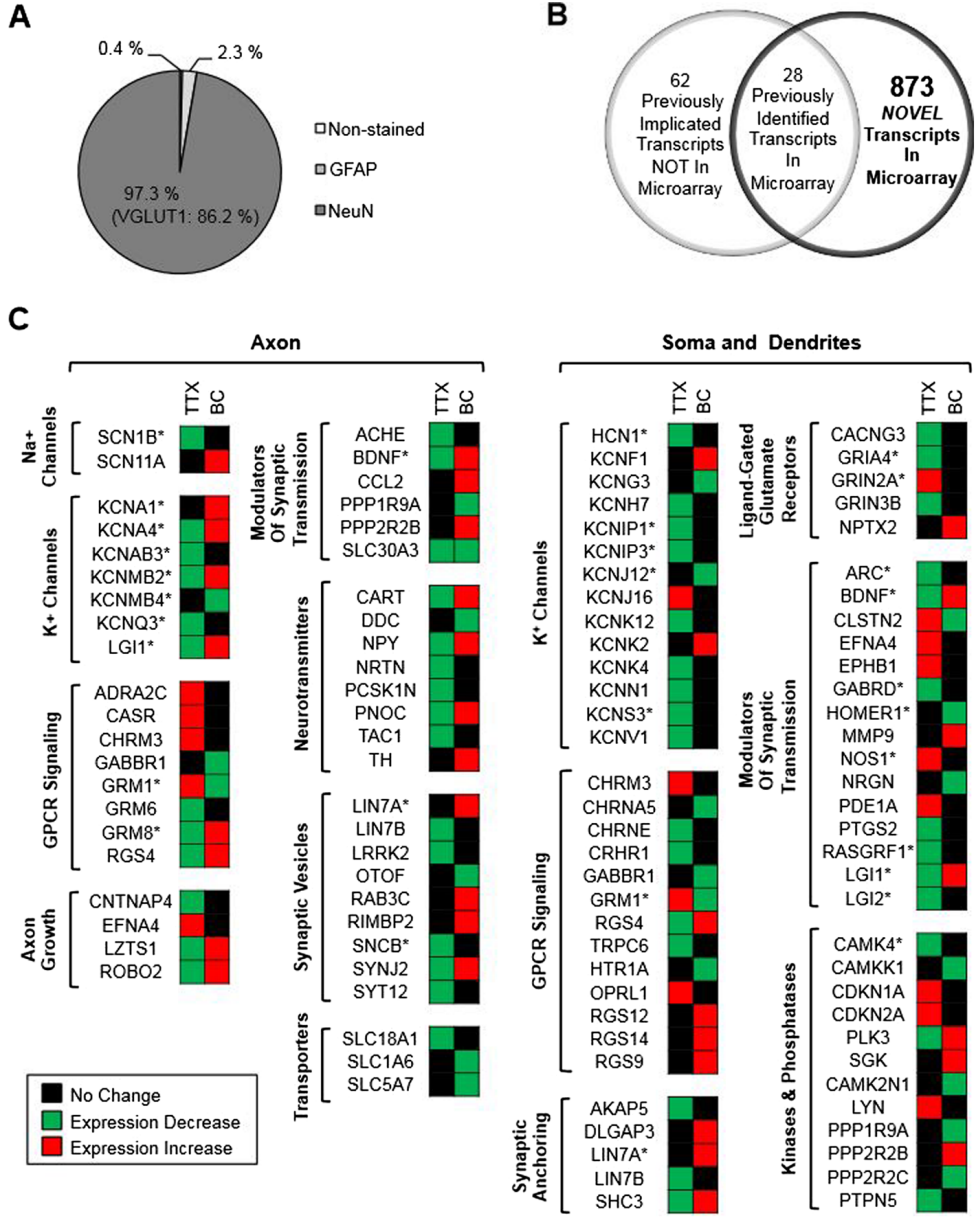
**Chronic activity alterations regulate genes involved in hippocampal synaptic and intrinsic membrane properties. (A)** Pie chart showing the relative proportion of neurons and glia in rat dissociated hippocampal neuronal culture. Immunostaining studies revealed that 2.3 ± 2.1% of cells expressed glial marker, glial fibrillary acid protein (GFAP) whereas 97.3 ± 2.9% were positive for the neuronal marker NeuN. Of the NeuN-positive cells, 86.2 ± 5.0% were immunoreactive for vesicular glutamate transporter 1 (VGLUT1) indicative of glutamatergic neurons. **(B)** Venn diagram showing 901 transcripts identified by microarray analysis whose expression were significantly changed by 48 h TTX and BC treatment compared to CTL-H_2_O treatment at 48 h using an FDR < 0.05 and a fold change < 0.667 (repressed) or > 1.5 (induced) (black circle). The genes whose protein products have previously been implicated in homeostatic plasticity are also indicated (gray circle). **(C)** The colormetric representation shows select genes, indicated by HUGO Gene Nomenclature gene symbols, whose transcripts were repressed (green) and induced (red). The *denotes genes that further tested for activity-dependent changes in their mRNA levels using QPCR analysis. Using Gene Ontology analysis, select activity-regulated genes were grouped by cellular components, molecular function, and biological processes.

Quadruplicate RNA samples for each treatment were subjected to cDNA microarray analysis (41,012 total gene probes). Gene expression profiling in high-density cultures composed primarily of glutamatergic neurons (Figure 2A) revealed 901 transcripts, which were significantly regulated by prolonged activity manipulation (Figure 2B). Consistent with previous studies reporting that alterations in intracellular Ca^2+^ concentration are required for homeostatic regulation of synaptic and intrinsic neuronal properties [2,11,13-15,18], we found that genes classified using the Gene Ontology (GO) terms “synaptic transmission”, “transmission of nerve impulses”, and “cytosolic Ca^2+^ ion homeostasis” were significantly over-represented in “chronic activity”-regulated gene transcripts (Table 3). The enriched GO classes also included development, behavior, and signaling pathways (Table 3), underscoring the complex impact that prolonged activity alteration exerts on gene regulation.

**Table 3:** **Enriched gene ontology (GO) categories identified in microarray analysis following 48 h pharmacological treatment**

**GO biological processes**	***P*** **-values**	
	**TTX**	**BC**
Cell-cell signaling	7.40e^−6^	1.60e^−4^
Transmission of nerve impulse	4.60e^−5^	2.00e^−4^
Synaptic transmission	1.00e^−5^	4.50e^−4^
Neuropeptide signaling pathway	4.80e^−5^	1.40e^−2^
Intracellular signaling cascade	5.70e^−9^	2.90e^−8^
Cytosolic Ca^2+^ ion homeostasis	4.90e^−8^	4.70e^−3^
Regulation of phosphorylation	1.00e^−5^	9.60e^−2^
Second-messenger-mediated signaling	1.30e^−5^	2.90e^−4^
cAMP-mediated signaling	4.60e^−3^	1.10e^−2^
Ion transport	4.90e^−4^	5.60e^−2^
K^+^ ion transport	2.40e^−5^	4.60e^−2^
Metal ion transport	2.10e^−4^	3.50e^−2^
Nervous system development	2.10e^−5^	1.10e^−2^
Neuron differentiation	7.70e^−4^	1.80e^−2^
Neuron development	8.20e^−4^	1.80e^−2^
Axon Guidance	N/A	9.30e^−3^
Behavior	1.80e^−7^	5.10e^−6^
Learning or memory	4.30e^−5^	3.30e^−4^
Regulation of synaptic plasticity	7.10e^−4^	N/A

Of these “chronic activity”-regulated gene transcripts, we identified 873 genes, which were not previously implicated in homeostatic plasticity (Figure 2B,C). Of these transcripts, the GO theme “K^+^ ion transport” was strikingly over-represented (Table 3). TTX treatment decreased mRNA levels for 14 of 15 K^+^ channel genes, whose protein products encode the principle and auxiliary subunits for K_v_1 channels (*KCNA4, KCNAB3* and *LGI1*), K_v_2.1 channels (*KCNS3* and *KCNV1*), K_v_4 channels (*KCNIP1* and *KCNIP3*), K_v_7 channels (*KCNQ3*), HERG channels (*KCNH7*), hyperpolarization-activated cyclic nucleotide-gated non-selective cation HCN channels (*HCN1*), Ca^2+^-activated large conductance BK channels (*KCNMB2*), Ca^2+^-activated small conductance SK channels (*KCNN1*), TRAAK leak K^+^ channels (*KCNK4*), and THIK2 leak K^+^ channels (*KCNK*12) (Figure 2C).

Conversely, BC treatment increased mRNA levels of genes whose protein products encode K_v_1 channels (*KCNA1*, *KCNA4, LGI1),* BK channels (*KCNMB2*), TREK1 leak K^+^ channels (*KCNK2)*, and delayed rectifier K^+^ channels (*KCNF1)* (Figure 2C). Transcripts of the genes that encode negative regulators of BK and K_v_2.1 channels (*KCNJ12*, *KCNG3*, and *KCNMB4*) were decreased by BC application (Figure 2C). Since K^+^ channels provide a main driving force for membrane hyperpolarization that dampens intrinsic excitability [9], these results suggest that down-regulation of multiple K^+^ channel genes may contribute to the TTX-induced rise in intrinsic excitability whereas BC-induced dampening of intrinsic excitability at 80–90 pA injections (Figure 1B) may be achieved in part by enhanced outward K^+^ current through K_v_1, K_v_2.1, or BK channels.

Our microarray analyses have also revealed novel synaptic genes whose protein products have not been implicated in homeostatic plasticity including *GRM8*, *LGI2*, *LIN7A*, *NOS1*, *SNCB*, and *GABRD* (Figure 2B,C). Of particular interest, neuronal nitric oxide synthase (nNOS) produces NO upon stimulation of NMDARs [19]. Since NO is required for the induction of long-term potentiation at excitatory synapses [20], TTX-induced *NOS1* expression (Figure 2C) could increase synaptic strength during the expression of homeostatic plasticity. Considering the potent roles of presynaptic mGluR8 in suppressing glutamate release in the hippocampus [21] as well as Lin7A and α-synuclein in synaptic vesicle exocytosis [22-25], the modulation of *GRM8*, *LIN7A*, and *SNCB* expression by chronic activity alteration (Figure 2C) may be involved in presynaptic expression of homeostatic synaptic scaling [13,26-28].

The chronic activity-regulated gene transcripts included 28 genes whose protein products have previously been implicated in homeostatic plasticity (Figure 2B), including *ARC* [29], *BDNF* [3,30-32], *NPTX2* [33], *GRIA4* [33], *HOMER1* [34], *RASGRF1* [35] and *CAMK4* [11,14]. Consistent with TTX-induced decreases in *ARC* and *HOMER1* mRNAs (Figure 2C), synaptic scaling induced by chronic inactivity is mediated by diminished Arc/Arg3.1 [29] and Homer1a [34]. Interestingly, although prolonged activity enhancement reduces the localization of RasGRF1 and surface GluA1 at the proximal dendrites of hippocampal cultured neurons [35], we discover that TTX but not BC treatment reduced *RASGRF1* mRNAs.

Not identified by our microarray were at least 62 transcripts whose protein products have previously been implicated in homeostatic plasticity (Figure 2B). Previous studies have reported that dendritic local protein synthesis is required for synaptic scaling induced by chronic treatment with TTX and APV [36] whereas prolonged inhibition of the ubiquitin proteasome system has been shown to mimic synaptic scaling induced by chronic activity blockade in cultured hippocampal neurons [37]. Recently, chronic inactivity-induced degradation of PICK1 is reported to enhance surface expression of GluA2-containing AMPARs during the expression of synaptic scaling in cultured cortical neurons [38]. These studies suggest that homeostatic plasticity involves additional posttranscriptional regulatory mechanisms that influence protein synthesis and degradation.

### Chronic inhibition of NMDARs drives a homeostatic increase in intrinsic excitability and down-regulation of K^+^ channel genes

**Figure 3:**
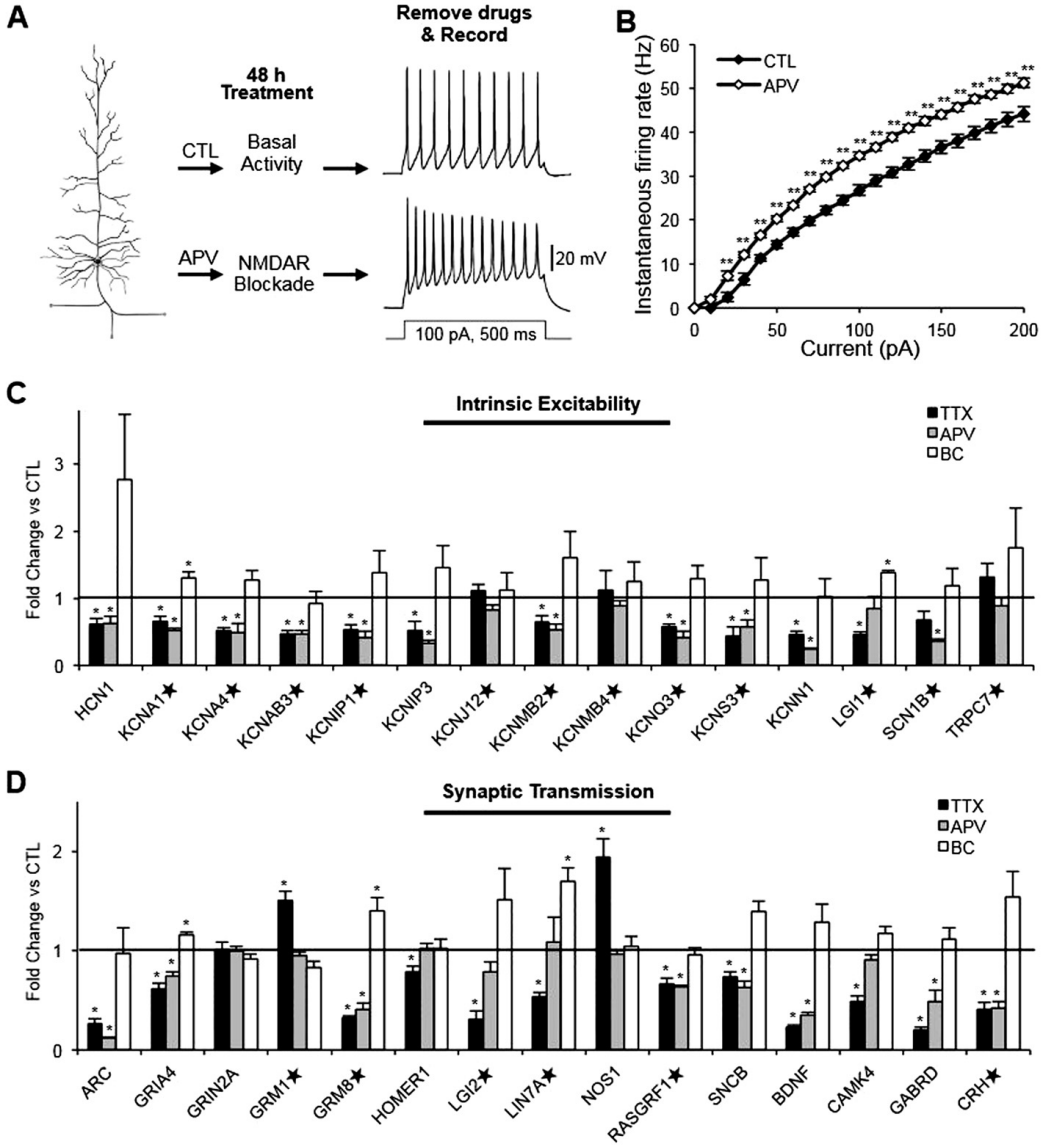
**Chronic activity blockade or prolonged NMDAR inhibition reduces gene expression of K** ^+^ **channels. (A,B)** Rat dissociated hippocampal neurons cultured at high density (DIV 12–14) were treated with the NMDAR antagonist APV (100 μM) or control (CTL-H_2_O, 0.1% H_2_O) for 48 h. **(A)** Representative spike trains are shown. **(B)** Average AP firing rates (Hz) measured in pyramidal neurons treated for 48 h with CTL-H_2_O (n = 9) or APV (n = 9) for 48 h. APV treatment significantly increased AP firing frequency compared to CTL-H_2_O treatment. **(C,D)** QPCR validation of select genes involved in intrinsic excitability **(C)** and synaptic transmission **(D)** identified by microarray analysis (n = 5 per treatment). The mRNA levels of all samples were normalized to the housekeeping gene GAPDH, which was unaffected by these treatments. Following normalization to GAPDH cDNA levels, the relative fold change for each treatment compared to reference control was determined and has been shown. Treatment with TTX or APV decreased the mRNA levels of most of the tested K^+^ channel genes. In contrast, BC application had no effect on most K^+^ channel genes except for *KCNA1* and *LGI1* which were up-regulated. The ★ denotes genes whose protein products have not previously been implicated in homeostatic plasticity. Mean ± SEM (*p < 0.05, **p < 0.01).

Ca^2+^ influx through either NMDARs or L-type VGCCs activates activity-dependent signaling cascades that regulate the activity of transcriptional regulators, which in turn modulate the expression of gene products important for neural development and plasticity [17]. We have previously reported that prolonged inhibition of NMDARs but not L-type VGCCs leads to a homeostatic increase in intrinsic excitability in low-density hippocampal neuronal culture [2]. Similarly, 48 h treatment with NMDAR antagonist APV (100 μM) significantly increased AP firing rates compared to CTL-H_2_O treatment for all current injections over 20 pA in hippocampal neurons cultured at high density (100 pA, CTL-H_2_O: 26.7 ± 1.6 Hz, APV: 34.6 ± 0.7 Hz, *p* < 0.01) (Figure 3A,B). 48 h APV treatment altered AP rise time, decay time, and half width of hippocampal neurons at 100 pA injection (Table 2), but did not affect their membrane capacitance and resting membrane potential (Table 1).

Similar to TTX treatment, 48 h APV application significantly increased the input resistance (Table 1) and decreased average rheobase current compared to CTL-H_2_O treatment (Additional file 2: Table S1). Since a change in input resistance could conceivably affect excitability, the AP firing rates were recalculated in response to current injections from rheobase to rheobase +100 pA as previously described [39]. These results revealed that 48 h TTX treatment increased AP firing frequency compared to CTL-H_2_O treatment for all current injections over 70 pA from the rheobase current (Additional file 3: Figure S2A, *p* < 0.05). Similarly, APV application significantly increased AP firing rates compared to CTL treatment for all current injections over 50 pA from the rheobase current (Additional file 3: Figure S2A, *p* < 0.05). Taken together, these findings indicate that chronic blockade of NMDAR increases the intrinsic excitability of hippocampal neurons, which is not dependent on an increase in input resistance.

The remarkably similar effect on AP firing rates elicited by either TTX or APV treatment (Figures 1, 3A,B, Additional file 3: Figure S2A) led us to postulate that similar gene cohorts were regulated by these treatments. Indeed, QPCR revealed that 48 h treatment with TTX or APV significantly decreased the mRNA levels of K^+^ channel genes including *HCN1*, *KCNA1, KCNA4, KCNAB3, KCNIP1, KCNIP3, KCNMB2, KCNQ3, KCNS3,* and *KCNN1* (Figure 3C). *LGI1* mRNA expression was reduced by TTX treatment but not APV treatment (Figure 3C). Consistent with our data that BC application for 48 h has little or no effect on AP firing rate (Figure 1), BC treatment did not alter the mRNA levels of most K^+^ channel genes except for *KCNA1* and *LGI1* which were increased (Figure 3C). Neither TTX nor BC treatment affected the mRNA level of Glyceraldhyde-3-phosphate dehydrogenase (*GAPDH*) gene, which serves as a control gene for these treatments. These results together suggest that transcriptional down-regulation of K^+^ channel genes by NMDAR signaling may mediate a homeostatic rise in intrinsic excitability in response to chronic activity blockade.

Although chronic TTX treatment has been shown to increase average amplitude of Na^+^ currents in dissociated cortical neurons [4], microarray analysis identified only two Na^+^ channel genes, *SCN1B* and *SCN11A*, that were regulated by prolonged activity manipulation (Figure 2C). QPCR verification revealed that *SCN1B* mRNA was significantly decreased by APV treatment (*p* < 0.05), but not TTX treatment (Figure 3C). *SCN1B* encodes the β1 regulatory subunit (Na_v_1β), which modulates current density and subcellular localization of voltage-gated Na^+^ channels [40]. Interestingly, Na_v_1β was also shown to bind to K_v_4.2 and increase current densities of K_v_4.2 channels [41]. Considering that impaired AP repolarization and enhanced repetitive firing of AP were observed in cortical pyramidal neurons of Na_v_1β-null mice [41], we speculate that a decrease in *SCN1B* expression and depletion of Na_v_1β subunits could contribute to the APV-induced increase in firing rates by down-regulating K_v_4.2 channels.

Consistent with our microarray, the QPCR analysis revealed that 48 h BC treatment up-regulated *GRIA4, GRM8,* and *LIN7A* whereas 48 h TTX treatment significantly induced *GRM1* and *NOS1* and repressed 12 genes involved in synaptic transmission including *ARC, GRIA4, GRM8, HOMER1, LGI2, LIN7A, RASGRF1, SNCB, BDNF, CAMK4, GABRD,* and *CRH* (Figure 3D). In contrast, only half of the genes involved in synaptic transmission selected for further verification were regulated similarly by APV and TTX (Figure 3D). These genes include *ARC*, *GRIA4*, *GRM8*, *RASGRF1*, *SNCB*, *BDNF*, *GABRD,* and *CRH* (Figure 3D). In cortical cultures, exogenous BDNF prevents the TTX-induced increase in AP firing rates, while scavenging endogenous BDNF mimics the increase in intrinsic excitability that occurs with chronic activity blockade [3,31]. Given that BDNF is released in an activity-dependent manner [42] and known to stimulate transcription [43,44], our results in hippocampal neurons is consistent with the hypothesis that BDNF serves as an upstream regulator of homeostatic intrinsic plasticity [1]. Furthermore, RasGRF1 knockout neurons display enhanced spontaneous neuronal activity and AP firing rates in hippocampal neurons [45], suggesting that a decrease in *RASGRF1* expression, which occurs with TTX (Figure 3D), may play a role in the homeostatic increase in intrinsic excitability.

### Chronic inhibition of L-type VGCCs does not affect intrinsic excitability or expression of most K^+^ channel genes

**Figure 4:**
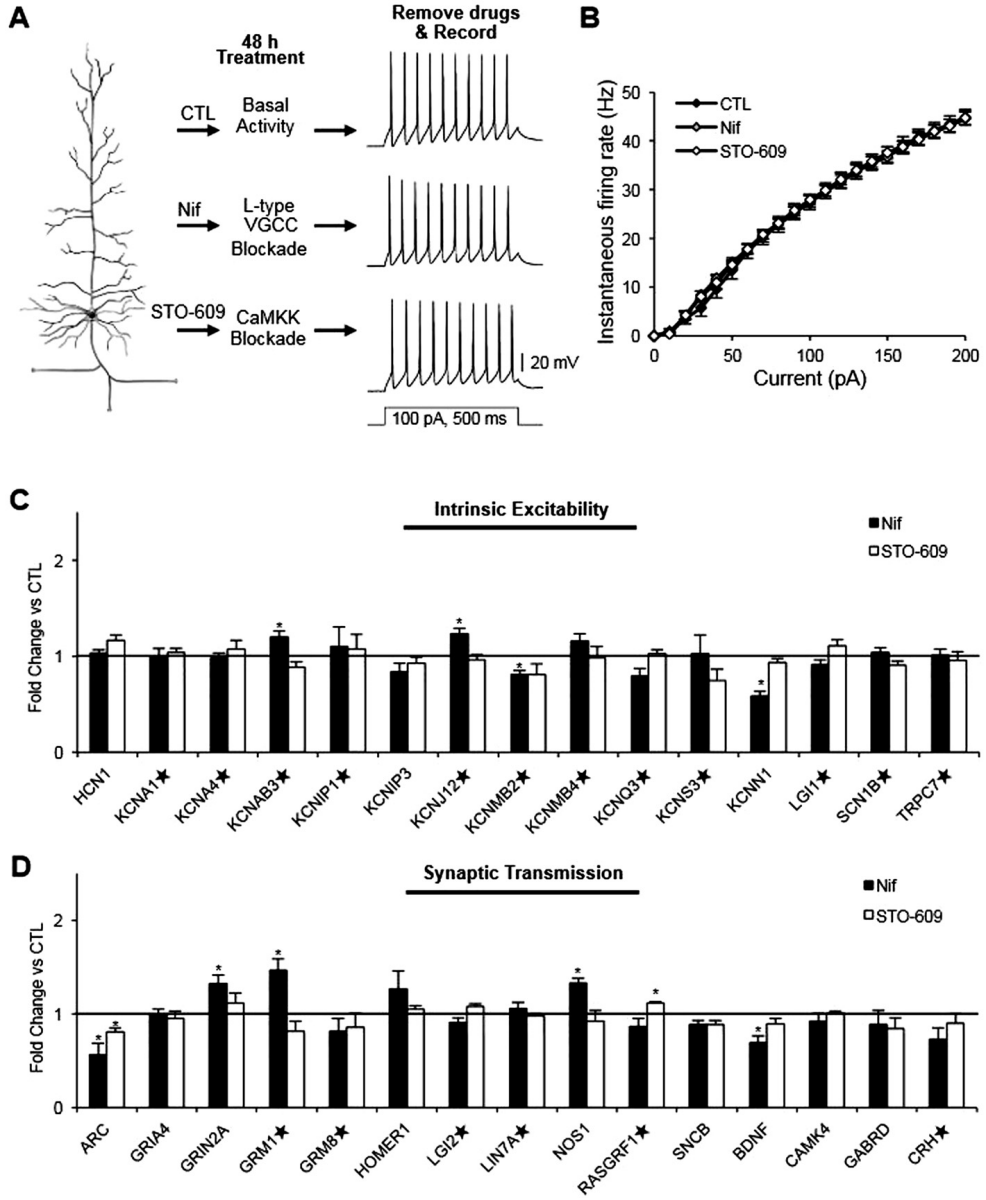
**Prolonged inhibition of L-type VGCCs alone does not affect intrinsic excitability or mRNA levels of most K** ^+^ **channel genes. (A,B)** Rat dissociated hippocampal neurons cultured at high density (DIV 12–14) were treated with L-type VGCC inhibitor Nif (20 μM), CaMKK inhibitor STO-609 (2 μM), or control (CTL-DMSO, 0.1% DMSO) for 48 h. **(A)** Representative spike trains are shown. **(B)** Average AP firing rates (Hz) measured in pyramidal neurons treated with CTL-DMSO (n = 12), Nif (n = 9), or STO-609 (n = 10) for 48 h. Nif or STO-609 treatment did not alter AP firing frequency compared to CTL-DMSO treatment. **(C,D)** QPCR validation of select genes that we identified by microarray analysis and have been implicated in intrinsic excitability **(C)** and synaptic transmission **(D)** identified by microarray analysis (n = 5 per treatment). Expression of intrinsic excitability genes was not altered by Nif or STO-609 treatment, except *KCNJ12, KCNMB2,* and *KCNAB3*. The ★ denotes genes whose protein products have not previously been implicated in homeostatic plasticity. Mean ± SEM (*p < 0.05).

Chronic blockade of either L-type VGCCs or the downstream target Ca^2+^/calmodulin-dependent protein kinase kinase (CaMKK) mimics TTX-induced synaptic scaling [14]. To investigate whether similar manipulations dictate AP firing frequency, we administered the L-type VGCC antagonist nifedipine (Nif, 20 μM), CaMKK inhibitor STO-609 (2 μM), or vehicle control (CTL-DMSO, 0.1% DMSO) for 48 h. Consistent with previous reports in low-density hippocampal neuronal culture [2], 48 h treatment with Nif or STO-609 did not alter AP firing frequency of hippocampal neurons cultured at high density for all current injections compared to CTL-DMSO treatment (100 pA, CTL-DMSO: 27.4 ± 1.5 Hz, 20 μM Nif: 27.5 ± 1.2 Hz, STO-609: 27.9 ± 1.0 Hz) (Figure 4A,B). Passive membrane properties as well as AP properties were unaffected by Nif or STO-609 treatment (Tables 1 and 2).

Importantly, 48 h inhibition of L-type VGCCs had little or no effect on the expression of the majority of intrinsic excitability genes tested, except for *KCNAB3, KCNJ12, KCNMB2*, and *KCNN1.* The mRNA levels of *KCNAB3* and *KCNJ12* were increased while those of *KCNMB2* and *KCNN1* were reduced (Figure 4C). Similarly, 48 h STO-609 treatment did not affect the expression of intrinsic excitability genes. Among synaptic genes, Nif treatment decreased *ARC* and *BDNF* transcripts and increased *GRM1* and *NOS1* transcripts (Figure 4D) in a manner that mimicked the altered gene expression induced by TTX at 48 h (Figure 3D). However, only *ARC* mRNA was reduced by STO-609 treatment at 48 h (Figure 4D). The minimal effect on either AP firing frequency or expression of K^+^ channel genes in response to 48 h Nif or STO-609 administration suggests that a decrease in L-type VGCC/CaMKK-dependent signaling is likely not the primary mediator of the homeostatic increase in intrinsic excitability induced by chronic activity blockade.

### Inhibition of NMDARs or L-type VGCCs during chronic activity blockade increases intrinsic excitability to the same extent as chronic activity blockade alone

**Figure 5:**
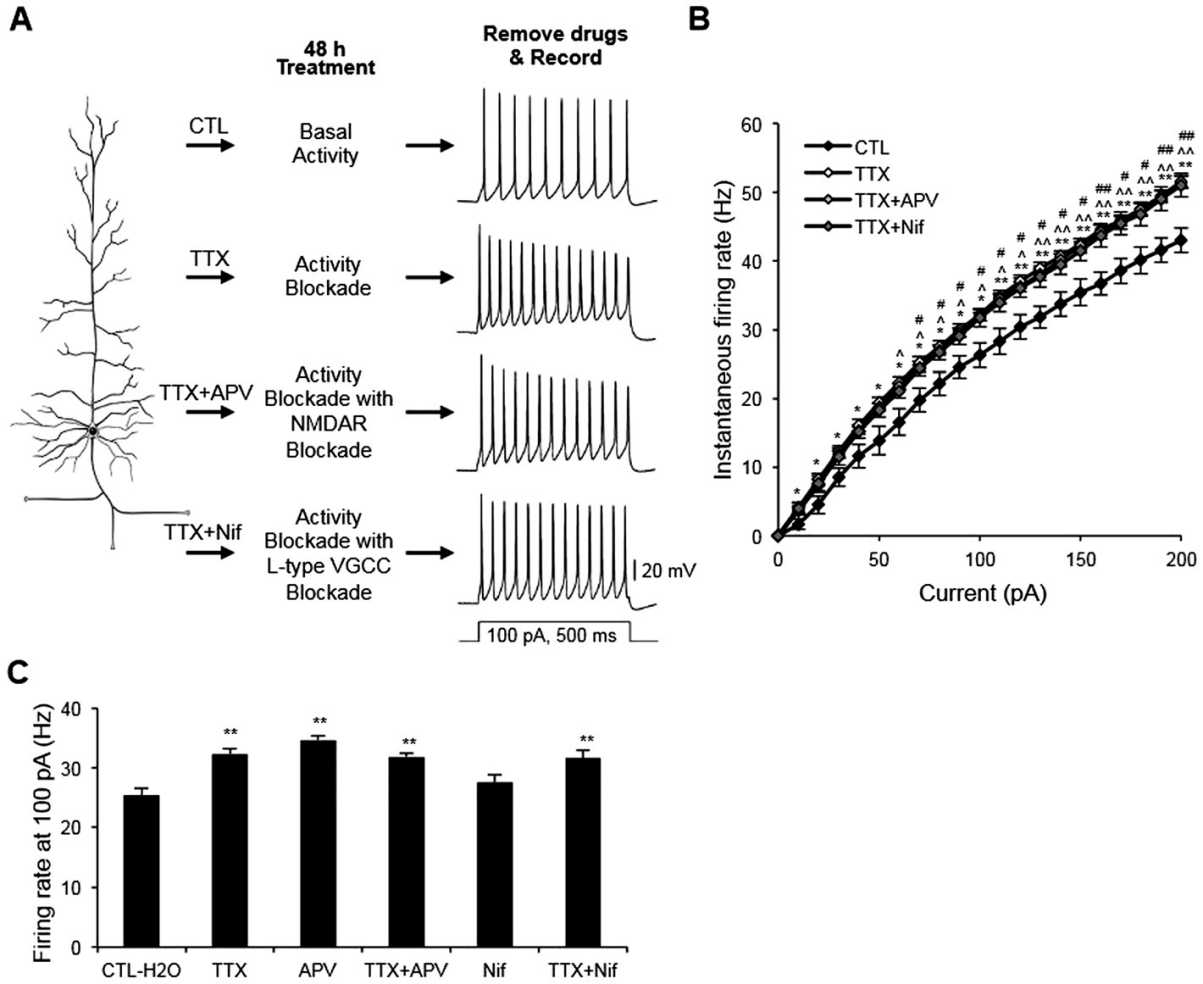
**Inhibition of NMDARs or L-type VGCCs during chronic activity blockade increases intrinsic excitability to the same extent as chronic activity blockade alone. (A,B)** Rat dissociated hippocampal neurons cultured at high density (DIV 12–14) were treated for 48 h with control (CTL-H_2_O, 0.1% H_2_O), TTX (0.5 μM), TTX (0.5 μM) + APV (100 μM), or TTX (0.5 μM) + Nif (20 μM). **(A)** Representative spike trains are shown. **(B)** Average AP firing rates (Hz) measured in pyramidal neurons treated for 48 h with CTL-H_2_O (n = 9), TTX (n = 10), TTX + APV (n = 10), or TTX + Nif (n = 9). Mean ± SEM (*p < 0.05, **p < 0.01 for CTL vs. TTX; ^*p* < 0.05, ^^*p* < 0.01 for CTL vs. TTX + APV; #*p* < 0.05, ##*p* < 0.01 for CTL vs. TTX + Nif). **(C)** A summary plot illustrating the effect of pharmacological treatments on instantaneous firing rates at 100 pA. Treatment of hippocampal neurons with TTX and co-treatment with either TTX and APV or TTX and Nif significantly increased AP firing frequency compared to CTL-H_2_O treatment. The average AP rates at 100 pA injection for APV and Nif treatments are from Figures 3B and 4B. Mean ± SEM (**p < 0.01).

To confirm that down-regulation of NMDAR activity but not L-type VGCC activity mediates the compensatory increase in AP firing rates induced by 48 h TTX treatment, we co-treated cultured hippocampal neurons for 48 h with TTX (0.5 μM) and APV (100 μM). Co-treatment with TTX and APV led to a marked enhancement in AP firing rates compared to CTL treatment for all current injections over 60 pA (100 pA, CTL-H_2_O: 26.2 ± 1.7 Hz, TTX + APV: 31.8 ± 0.5 Hz, *p* < 0.05) (Figure 5A,B). Importantly, we recorded similar AP firing rate increases in neurons treated for 48 h with TTX alone (Figure 5A,B) or APV alone (Figure 3A,B). At 100 pA current injection, the mean AP firing frequency in neurons co-treated with TTX and APV was indifferent from that in TTX-treated neurons and APV-treated neurons (*p* > 0.05) (Figure 5C), further supporting our finding that decreased NMDAR activity contributes to elevated intrinsic excitability following chronic activity blockade.

We demonstrate that 48 h TTX treatment alone significantly increased AP firing rates (Figure 1, 5A,B, Additional file 3: Figure S2A) whereas Nif treatment alone did not (Figure 4A,B, Additional file 3: Figure S2B). Importantly, co-treatment with TTX and Nif increased the firing rates compared to CTL treatment for all current injections over 70 pA (Figure 5A,B). The mean AP firing frequency in neurons co-treated with TTX and Nif at 100 pA current injection was not different from that in TTX-treated neurons (Figure 5C, TTX + Nif: 31.6 ± 1.3 Hz; TTX: 32.3 ± 0.8 Hz, *p* > 0.05), which provides evidence that chronic inhibition of L-type VGCC activity alone is not responsible for the homeostatic increase in AP firing rates.

Given that blockade of neuronal activity or inhibition of NMDARs initially decreases Ca^2+^ influx [14,46-48], our findings suggest that a reduction in intracellular Ca^2+^ influx through NMDAR but not L-type contributes to increased intrinsic excitability following chronic activity blockade. This hypothesis is further supported by the fact that only half of the genes involved in synaptic transmission, including *GRM1*, *HOMER1, and CAMK4* (Figure 3D), were down-regulated by TTX treatment but not APV treatment, although both treatments increased AP firing rates (Figures 1, 3A,B, and 5). These results suggest that the increase in intrinsic excitability induced by chronic TTX treatment is not dependent on group I mGluR-Homer1a signaling and CaMK4 signaling, despite the crucial roles for these signaling pathways in synaptic scaling [14,34]. In addition, 48 h TTX or Nif treatment but not APV treatment significantly increased the mRNA levels of *NOS1* (Figures 3D, 4D), a known regulator of hippocampal long-term potentiation [20]. Considering that prolonged inhibition of L-type VGCC mimics TTX-induced scaling of excitatory synapses [14] and not AP firing rates (Figures 4A,B, and 5), our results suggest that enhanced *NOS1* expression and NO signaling may play a role in synaptic scaling, but it is unlikely to increase intrinsic excitability upon chronic activity blockade.

Although these results are consistent with previous studies reporting that NMDAR activity does not mediate homeostatic synaptic plasticity in cortical neurons [13,14,49-51], inhibition of NMDAR-mediated Ca^2+^ influx was recently implicated in synaptic scaling [46]. Kim and Ziff showed that TTX application acutely decreases intracellular Ca^2+^ concentration in cultured cortical neurons, which was restored by 48 h via upregulation of synaptic Ca^2+^-permeable AMPARs [46]. Since APV treatment also causes an initial decline of somatic Ca^2+^ concentration, the authors suggested that the restoration of Ca^2+^ levels was initiated by reduced NMDAR activity upon TTX treatment [46]. Consistent with the importance of restoring Ca^2+^ signaling in homeostatic plasticity, we discovered that 48 h TTX treatment leads to significant changes in transcripts implicated in “cytosolic Ca^2+^ ion homeostasis” (Table 3). Thus, additional studies are needed to identify the activity sensor and dissect the underlying signaling pathways responsible for synaptic scaling induced by chronic activity blockade in hippocampal neurons.

### Chronic activity blockade or chronic NMDAR inhibition reduces total K^+^ current, K_v_1 current and K_v_7 current

**Figure 6:**
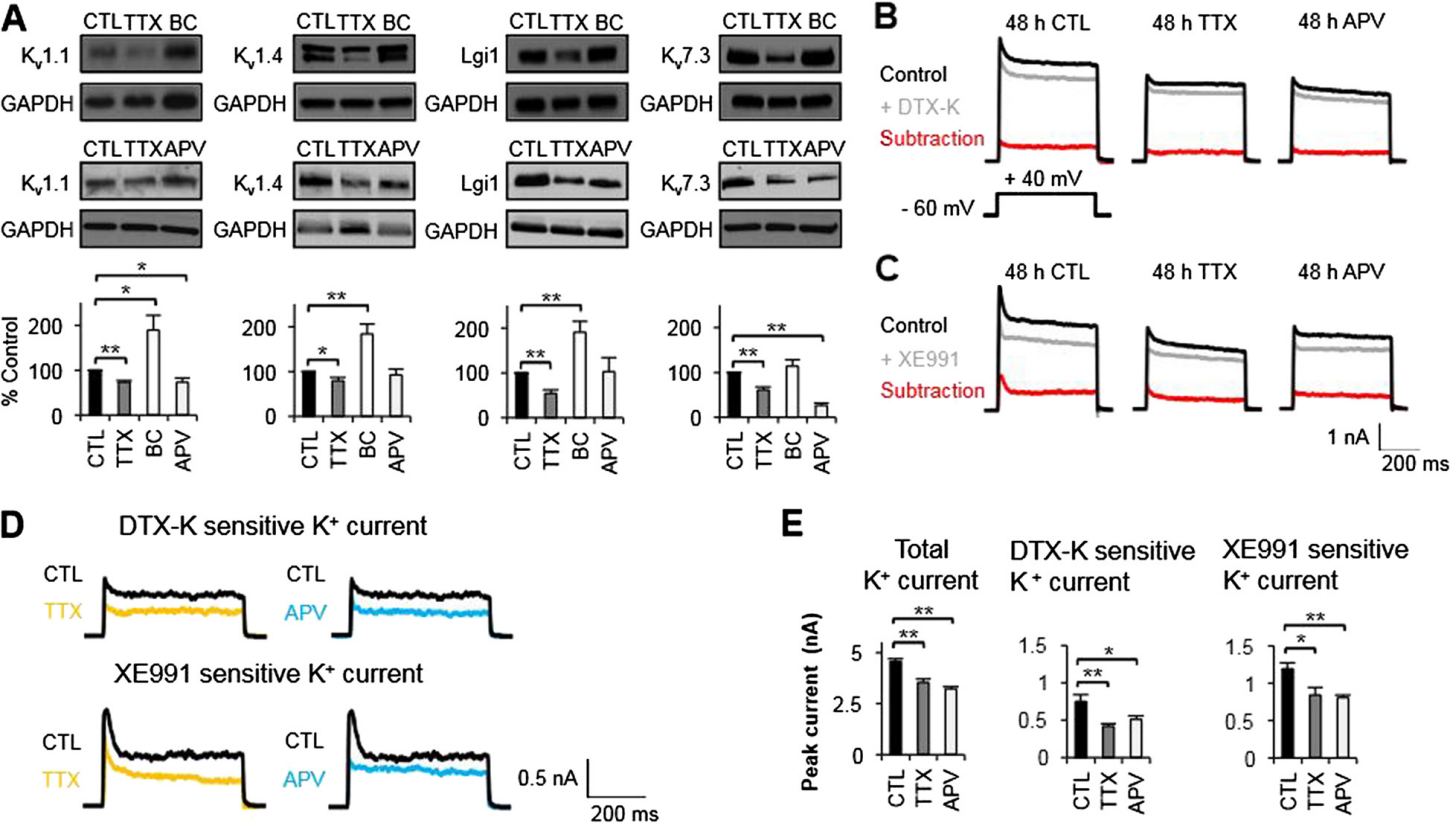
**Chronic activity blockade or prolonged NMDAR inhibition reduces protein and current expression of K** ^+^ **channels. (A)** Immunoblot analyses confirming activity-dependent modulation of the principle and auxiliary subunits of K_v_1 channels (K_v_1.1, K_v_1.4, Lgi1) and K_v_7/KCNQ channels (K_v_7.3) following 48 h application of TTX or BC (n = 6–12 per treatment). The level of each protein was normalized to GAPDH, followed by normalization to CTL-H_2_O. **(B-E)** Voltage clamp recordings of K^+^ currents evoked by depolarizing voltage step to +40 mV in cultured hippocampal neurons (DIV 12–14) following 48 h treatment with CTL, TTX or APV. After recording total K^+^ currents under vehicle control (0.1% H_2_O), dendrotoxin-K (DTX-K, 100 nM) or XE991 (10 μM) were applied and recordings of K^+^ currents from the same neurons were repeated. **(B,C)** Representative traces of K^+^ currents before (top, black) and after (middle, gray) application with K_v_1 channel specific antagonist, DTX-K **(B)** or K_v_7/KCNQ channel specific antagonist, XE991 **(C)**. The DTX-K- and XE991-sensitive currents were isolated by subtraction (bottom, red). **(D)** Enlargement of the traces from DTX-K-and XE991-sensitive K^+^ currents in neurons treated for 48 h with CTL (black), TTX (orange), or APV (blue). **(E)** Summary plots illustrating the effect of 48 h treatment with CTL (n = 21), TTX (n = 22), or APV (n = 22) on total K^+^ currents and DTX-K-and XE991-sensitive K^+^ currents at +40 mV. **(A,E)** Mean ± SEM (*p < 0.05, **p < 0.01).

Our microarray analysis and QPCR verification revealed that 48 h treatment with TTX or APV significantly decreased the mRNA levels of K_v_1 and K_v_7 channels (Figures 2C and 3C) that are critical mediators of excitability in hippocampal neurons [52-57]. To test if the reduction in the K^+^ conductance mediated by these channels may contribute to homeostatic intrinsic plasticity, we first performed immunoblot analyses to examine the changes in K_v_1 and K_v_7 expression. TTX treatment reduced whereas BC application enhanced expression of K_v_1.1, K_v_1.4, and Lgi1 (Figure 6A), the protein products of *KCNA1*, *KCNA4* and *Lgi1* (Figure 3C). NMDAR inhibition also decreased K_v_1.1 expression (*p* < 0.05) (Figure 6A).

K_v_1 current critically regulates AP repolarization and wave form-modulation [53,57-60]. Specifically, K_v_1.1 and K_v_1.4 are found predominantly located to axons and generate fast activating and inactivating A-type K^+^ current and fast activating and slowly inactivating D-type K^+^ current [9,58,61]. The epilepsy-associated secreted protein Lgi1 selectively prevents K_v_1 channel inactivation mediated by K_v_β1 [62-64]. A-type and D-type K^+^ currents have been shown to decrease rapidly in response to acute activity enhancement. For example, induction of chemical long-term potentiation in hippocampal neuronal culture reduces the A-type K^+^ current [65]. Similarly, a conditional AP train at 10 Hz for 2 s was shown to accompany an immediate reduction in the D-type K^+^ current in hippocampal CA3 neurons [66].

To assess whether decreased expression of K_v_1.1, K_v_1.4, and Lgi1, which occurs upon chronic activity blockade (Figure 6A), affects K_v_1 current, voltage-clamp recordings were performed to isolate K_v_1 currents using K_v_1 channel specific inhibitor, dendrotoxin-K (DTX-K) (Figure 6B,D,E). In hippocampal neurons cultured at high density, the DTX-K-sensitive K_v_1 currents were significantly decreased upon 48 h application of TTX (+40 mV, 0.41 ± 0.03 nA, *p* < 0.01) or APV (+40 mV, 0.50 ± 0.04 nA, *p* < 0.05) compared to CTL-H_2_O treatment (+40 mV, 0.74 ± 0.09 nA, *p* < 0.01) (Figure 6B,D,E). Whereas down-regulation of D-type K^+^ current via K_v_1.2 internalization was observed upon acute enhancement of somatic activity in hippocampal CA3 neurons to mediate long-term potentiation of intrinsic excitability [66], our findings suggest that a decrease in K_v_1 current could contribute to a homeostatic increase of AP firing frequency in response to chronic activity blockade or prolonged inhibition of NMDARs.

TTX or APV treatment also reduced expression of K_v_7.3 (*p* < 0.05) (Figure 6A), the subunit encoded by *KCNQ3* (Figure 3C). Although neurons express K_v_7.2 - K_v_7.5 [67], K_v_7 channels composed of K_v_7.2 and K_v_7.3 produce slowly activating and non-inactivating M-type K^+^ current, which potently suppresses repetitive AP firing [52,55,56,68]. To test if TTX or APV treatment for 48 h decreases K_v_7 current, voltage-clamp recordings were repeated to isolate K_v_7 currents using K_v_7/KCNQ channel specific antagonist, XE991 (Figure 6C-E). TTX or APV treatment significantly reduced the XE991-sensitive K_v_7 currents (+40 mV, TTX: 0.83 ± 0.09 nA, APV: 0.80 ± 0.03 nA) compared to CTL-H_2_O treatment (+40 mV, 1.18 ± 0.08 nA, *p* < 0.05) (Figure 6C-E). Sustained excitation has been shown to potentiate hippocampal K_v_7 function and spike frequency adaptation [69]. Conversely, our results suggest that reduction in K_v_7 current and K_v_7.3 (Figure 6), which is the dominant subunit that targets KCNQ channel to the axonal initial segment [70-72], may contribute to homeostatic stabilization of AP firing in response to chronic activity blockade or prolonged inhibition of NMDARs. Interestingly, XE991-sensitive current in APV-treated neurons became non-inactivating compared to that of TTX-treated neurons (Figure 6D). Though highly speculative, this could be due to the possibility that APV treatment may down-regulate K_v_7.4 and/or K_v_7.5 subunits that mediate inactivation [73].

Furthermore, TTX or APV treatment also reduced total K^+^ current (Figure 6B,C,E), which is consistent with our results that these treatments down-regulate multiple K^+^ channel transcripts (Figures 2C and 3C). For example, TTX or APV treatment decreased *HCN1* and *KCNMB2* mRNAs*,* which encode HCN channels and BKβ2, respectively. Enriched dendritically, HCN channels mediate activity-dependent h-current (*I*_h_) [74-76], which attenuates summation of excitatory postsynaptic potentials [77,78]. Thus, a decrease in *HCN1* expression, which occurs with TTX (Figures 2C and 3C), could enhance dendritic excitability. In hippocampal neurons, inactivation of BK channels contributes to frequency-dependent spike broadening [79]. Given that BKβ2 promotes inactivation of BK channels [80], a reduction in BKβ2 could potentiate intrinsic excitability by regulating AP repolarization and spike afterhyperpolarization [53,81,82]. Reduction in total K^+^ current by 48 h TTX or APV treatment (Figure 6B,C,E) is also consistent with our findings that these treatments significantly increase the input resistance of hippocampal neurons without altering their resting membrane potential (Table 1). These changes are most likely caused by the closure of ion channels, especially K^+^ channels. These results together suggest that a homeostatic increase in AP firing upon prolonged activity blockade may be achieved, in part, by NMDAR-dependent down-regulation of multiple K^+^ channels including K_v_1 and K_v_7 channels.

### Homeostatic intrinsic plasticity in high-density versus low-density hippocampal neuronal culture

We have previously reported that BC treatment for 48 h leads to a reduction in AP firing rates compared to CTL treatment in dissociated hippocampal neurons cultured at low density [2]. However, we did not observe the same effect in age-matched, high-density cultures (Figure 1). Since hippocampal neurons in high-density cultures are more excitable than those in low-density cultures (Additional file 4: Figure S3, Additional files 5 and 6: Tables S2 and S3), we speculate that inhibition of GABAergic transmission in hippocampal neurons cultured at high density may be insufficient to increase network activity required for the induction of homeostatic intrinsic plasticity.

Although TTX treatment for 48 h caused a homeostatic increase in AP firing rates in both high- and low-density cultures (Figure 1) [2], the same treatment reduced total K^+^ current in hippocampal neurons cultured at high density (Figure 6) but not those in low-density culture [2]. This could be due to the fact that CTL-treated, high-density cultures displayed larger total K^+^ current (Figure 6) than low-density cultures [2], enabling us to detect its significant reduction upon TTX treatment. Although we cannot exclude the possibility that ionic conductance mediating TTX-induced homeostatic intrinsic plasticity may not be equivalent in both high- and low-density cultures, our findings (Figure 6) are consistent with previous studies reporting that the elevation in AP firing frequency, which occurs with TTX treatment, is coupled with reduced K^+^ current density in dissociated cortical neurons [4].

**Figure 7:**
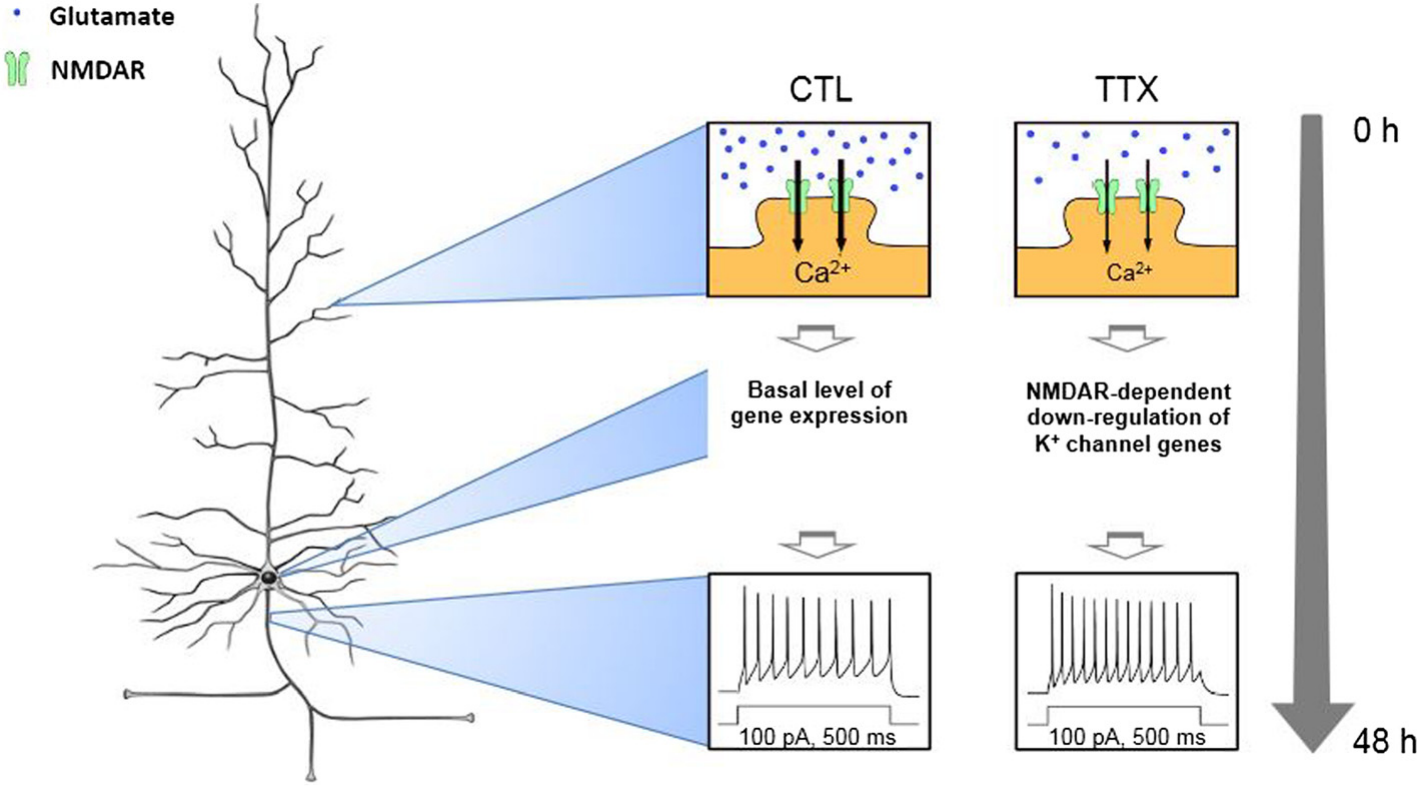
**Model for K** ^+^ **channel regulation during induction of homeostatic intrinsic plasticity.** TTX application rapidly blocks spontaneous action potential firing, causing an initial decrease in Ca^2+^ influx through NMDARs in dissociated hippocampal neurons cultured at high density. The subsequent transcriptional down-regulation of multiple K^+^ channel genes leads to a reduction in the protein expression of K^+^ channels at the neuronal plasma membrane. By 48 h, a decrease in currents through these K^+^ channels regulates AP and passive properties of hippocampal neurons, resulting in a homeostatic increase in the AP firing rates. The representative AP traces in the insets are from Figure 1A.

Despite the wide application of dissociated neuronal culture to study homeostatic plasticity [2-4,13,15,29-31,34,83], the results from this culture system should be interpreted with caution due to the dependence of homeostatic plasticity expression on culture density and age [28,84]. Remarkably, TTX and APV treatments but not Nif or STO-609 treatments led to a homeostatic increase in AP firing rates in both high-density (Figures 1, 3 and 4) and low-density cultures [2]. Importantly, co-treatment of neurons with either TTX and APV or TTX and Nif did not increase AP firing frequency to a greater extent than TTX treatment alone (Figure 5). Thus, regardless of culture density, homeostatic intrinsic plasticity induced by chronic activity blockade is most likely mediated by reduced activity of NMDARs and not L-type VGCCs (Figure 7).

## Conclusions

We have identified a novel NMDAR-mediated K^+^ channel-rich gene network dynamically regulated during homeostatic intrinsic plasticity in hippocampal neurons cultured at high density. Our findings support a critical role for the transcriptional down-regulation of a variety of K^+^ channels in the stabilization of hippocampal neuronal excitability upon chronic activity blockade (Figure 7). In contrast to a homeostatic increase in synaptic strength mediated by reduced activity of L-type VGCCs during chronic activity blockade [14], our findings indicate that the level of Ca^2+^ influx through NMDARs serves as a critical activity sensor capable of initiating transcriptional regulation of genes crucial for homeostatic intrinsic plasticity (Figure 7). We propose that homeostatic intrinsic plasticity induced by chronic activity blockade is achieved in 3 steps: (1) inhibition of neuronal activity initially decreases Ca^2+^ influx through NMDARs [14,46-48]; (2) transcriptional down-regulation of multiple K^+^ channel genes decreases expression of K^+^ channels; and (3) reduced current through these K^+^ channels regulates AP and passive properties of hippocampal neurons to increase AP firing rates. In addition to K^+^ channels, this study reveals a number of previously unknown targets of homeostatic plasticity, including genes that have been implicated in the regulation of synaptic strength by influencing release probability and postsynaptic receptor function [19-25,29-34] as well as maintaining Ca^2+^ homeostasis [46]. Future studies should explore how activity-dependent genes work together to simultaneously induce synaptic scaling and homeostatic intrinsic plasticity. Given that homeostatic intrinsic plasticity is hypothesized to contribute to hyperexcitability-associated neuropathologies [16,18,85], deciphering the activity-dependent transcriptional regulation of K^+^ channels during homeostatic intrinsic plasticity may also reveal novel therapeutic targets for these diseases.

## Materials and methods

### Materials

Chemical reagents used included tetrodotoxin, bicuculline, DL-2-amino-5-phosphonopentanoic, Nifedipine, STO-609 acetate, XE991 dihydrochloride (all Tocris), and dendrotoxin-K (Alomone). Antibodies used included anti-Lgi1 (Abcam), anti-GAPDH (Cell Signaling), anti-K_v_1.1 (Millipore), anti-K_v_1.1, anti-K_v_1.4 (Neuromab), and anti-KCNQ3 (Alamone, or a kind gift from Dr. E. Cooper, Baylor College of Medicine, Houston, Texas).

### Hippocampal neuronal culture

All experimental procedures involving animals were approved by the Institutional Animal Care and Use Committee at the University of California San Francisco and the University of Illinois Urbana-Champaign. Primary dissociated hippocampal cultures were prepared from E18.5 Sprague–Dawley rat embryos as described [2] and plated at high density (330 cells/mm^2^) onto poly L-lysine-coated (0.1 mg/mL) 12 mm glass coverslips (1.5×10^5^ cells/coverslip), or 30 or 60 mm cell culture dishes (6.5 ×10^5^ or 2.3×10^6^ cells/dish, respectively). At 10–13 days *in vitro* (DIV), neurons were treated for 48 h with (CTL-H_2_O, 0.1% H_2_O), (CTL-DMSO, 0.1% DMSO), TTX (0.5-1 μM), BC (20 μM), APV (100–200 μM), Nif (20 μM), or STO-609 (2 μM).

### RNA isolation and microarray analysis

Following pharmacological treatment, total RNA was isolated using an RNeasy kit (Qiagen) and stored at −80°C. Total RNA quality and quantity was assessed using a Pico Chip on an Agilent 2100 Bioanalyzer and Nanodrop ND-100 spectrophotometer. Quadruplicate RNA samples for each treatment were submitted to the UCSF Sandler Center Functional Genomics Core Facility for cDNA microarray hybridization to whole rat genome 4x44K Ink-jet arrays (Agilent, 41,012 total gene probes). Subsequent data extraction and analysis were performed at the core facility to determine the quantile normalization [86], B statistic [87], false discovery rate (FDR) [88] using the R package limma in Bioconductor [89,90]. Gene probes with an FDR < 0.05 and fold change of ≤ 0.667 (repressed) or ≥ 1.5 (induced) were considered differentially regulated. Gene ontology was analyzed using DAVID bioinformatics resources (http://david.abcc.ncifcrf.gov) [91] and primary literature searches.

### QPCR

To synthesize cDNA, reverse transcription was performed using total RNA (1–2 μg), random nanomers (15 μM), dNTPs (25 mM), M-MuLV reverse transcriptase (200 U/μL), and RNase inhibitor (40 U/μL)(New England Biolabs). The resulting cDNA was subjected to QPCR using the StepOnePlus real-time PCR system (Applied Biosystems) and SYBRGreenI (Invitrogen) to enable real-time quantification as described [92] with primers homologous to cDNAs corresponding to 31 genes of interest and *GAPDH* control gene (Additional file 7: Table S4). Primer efficiency was within 90-110% on a standard curve (slope of −3.30 = 100% optimal efficiency), with an R^2^ correlation coefficients > 0.99, and threshold cycle (Ct) values > 8 and < 35. The QPCR product specificity was confirmed through melt curve analysis. Following normalization to *GAPDH* cDNA levels, which is reflected in the ΔCt values, the relative quantification (RQ) of the fold change for each treatment compared to reference control was determined using the following equations: RQ = 2^(−ΔCt)^ / 2^(−ΔCt reference)^.

### Immunoblot analysis

Following pharmacological treatment, hippocampal cultured neurons were lysed in RIPA buffer and the resulting lysate samples were subjected to immunoblot analysis as described [93]. Densitometric quantification, following normalization to GAPDH was performed with ImageJ software (National Institutes of Health) as described [93].

### Whole cell patch clamp recordings

Whole-cell patch clamp recordings of AP firing and K^+^ currents were carried out at 23-25°C in neurons held at −60 mV as previously described [2] using a Multiclamp 700B amplifier, Digidata 1440A, and the pClamp 10.2 (Molecular Devices). Recordings were filtered at 2 kHz and digitized at 10 kHz. Recording analyses were performed using Clampfit 10.2 (Molecular Devices). Current clamp recordings of spontaneous AP firing were carried out as previously described [2] immediately after applying vehicle control (0.1% dH2O), TTX (0.5-1 μM), or BC (20 μM) in external solution containing (in mM): 126 NaCl, 3 KCl, 2 CaCl_2_, 2 MgSO_4_, 1 NaH_2_PO_4_, 25 NaHCO_3_ and 14 Dextrose, bubbled with 95% O_2_ and 5% CO_2_ (pH 7.4, 305–315 mOsm). Recording pipettes had a resistance of 3–5 MΩ when filled with internal solution containing (in mM): 130 KMeSO_4_, 10 KCl, 10 HEPES/K-HEPES, 2 MgSO_4_, 0.5 EGTA and 3 ATP (pH 7.3, 285–295 mOsm). Spontaneous firing was measured upon delivering zero current (0 pA) pulses.

To examine the induction of homeostatic intrinsic plasticity, current clamp recordings of AP were carried out immediately after the removal of 48 h TTX or BC treatment in external solution in the presence of the fast synaptic transmission blockers CNQX (20 μM) and APV (100 μM). AP firing rates (Hz) were measured upon delivering constant current pulses of 500 ms in the range 0 to 200 pA, and were averaged from 3 to 5 individual sweeps per each current injection. Neurons were omitted if the resting membrane potential was > −50 mV, or if no APs were discharged. Rheobase was defined as the minimal current that elicited at least one spike by incremental 10 pA current steps for 500 ms duration at a holding potential of −60 mV [39].

Voltage-clamp recordings of K^+^ currents were performed in external solution containing CNQX (20 μM) and APV (100 μM) to block the fast synaptic transmission as well as TTX (0.5 μM) and CdCl_2_ (400 μM) to block the Na^+^ and Ca^2+^ currents, respectively. Depolarizing steps (500 ms) were applied every 10 s intervals in +10 mV increments up to +40 mV. After recording K^+^ currents under vehicle control (0.1% H_2_O), the neurons were exposed to XE991 (10 μM) or dendrotoxin-K (DTX-K, 100 nM) and recordings of K^+^ currents were repeated. The XE991-sensitive K_v_7 currents or DTX-K-sensitive K_v_1 currents were computed by subtracting the K^+^ currents recorded in the presence of the inhibitors from the K^+^ currents recorded under the control condition.

### Statistical analyses

All data shown represent the mean value ± SEM. The number of separate neuronal culture dishes, RNA samples, and cells patched for electrophysiological studies are expressed as sample size *n.* Statistical analyses were performed with Microsoft Excel and Origin 9.1 (Origin Lab). Following Student’s t test or ANOVA, a priori value (*p*) < 0.05 was considered statistically significant.

## Additional files

Additional file 1: Figure S1. Effects of acute TTX or BC treatment on spontaneous firing of hippocampal pyramidal neurons. (A) Whole-cell patch clamp recording of rat dissociated hippocampal neurons cultured at high density (DIV 12–14) revealed that acute application of TTX (0.5 μM) rapidly blocks spontaneous firing of AP whereas acute application of BC (20 μM) leads to burst firing compared to CTL-H_2_O treatment. Additional file 2: Table S1. The average rheobase current of hippocampal pyramidal neurons cultured at high-density following 48 h pharmacological treatment. Rheobase (the minimal current that elicited at least one spike) was determined by incremental 10 pA current steps for 500 ms duration at a holding potential of −60 mV. Each value represents the mean ± SEM (***p* < 0.01 for CTL-H_2_O vs. APV). Additional file 3: Figure S2. The AP firing rates in response to current injections from rheobase to rheobase + 100 pA. Average AP firing rates (Hz) shown in Figures 1, 3 and 4 were recalculated in response to current injections from rheobase to rheobase +100 pA. (A) Treatment of hippocampal neurons for 48 h with TTX (n = 10) or APV (n = 9) significantly increased AP firing frequency compared to CTL-H_2_O treatment (n = 22). (B) Treatment for 48 h with Nif (n = 9) or STO-609 (n = 10) did not alter AP firing frequency compared to CTL-DMSO treatment (n = 12). (C) BC application for 48 h (n =9) did not change AP firing frequency compared to CTL-H_2_O application (n = 22). Mean ± SEM (**p* < 0.05, ***p* < 0.01 for CTL vs. TTX; ^*p* < 0.05, ^^*p* < 0.01 for CTL vs. APV). Additional file 4: Figure S3. Effect of culture density on innate excitability of hippocampal pyramidal neurons. (A,B) Whole-cell patch clamp recording of rat dissociated hippocampal neurons cultured at high density and low density (DIV 12–14) after 48 h application of vehicle control (CTL, 0.1% H_2_O). Following treatment removal, spike trains were evoked in pyramidal neurons by delivering constant somatic current pulses for 500 ms duration at a resting potential of −60 mV. (A) Representative spike trains are shown. (B) Average AP firing rates (Hz) were measured in pyramidal neurons cultured at high density (n = 22) and low density (n = 22). Mean ± SEM (**p* < 0.05, ***p* < 0.01 for CTL-LD vs. CTL-HD). Additional file 5: Table S2. Passive properties of hippocampal pyramidal neurons cultured at low density. *n*, number; *C*m, Whole-cell membrane capacitance; *R*in, input resistance; *V*m, resting membrane potential. Each value represents the mean ± SEM. Additional file 6: Table S3. AP properties of hippocampal pyramidal neurons cultured at low density. *V*T, voltage threshold for action potential; AP, Action potential; rise, 10-90% rise time of AP; decay, 10-90% decay time of AP; HW, half-width; fAHP, fast after-hyperpolarization. AP properties were measured from the first action potential evoked by a current step to 100 pA at a holding potential of −60 mV. Each value represents the mean ± SEM (**p* < 0.05 for CTL-LD vs. CTL-HD). Additional file 7: Table S4. Primer pairs for QPCR analysis. Primers were designed with Primer3 software (Whitehead Institute). Primer efficiency was within 90-110% on a standard curve (slope of −3.30 = 100% optimal efficiency), with an R2 correlation coefficients > 0.99, and threshold cycle (CT) values > 8 and < 35.

## Abbreviations

AP: Action potential
APV: DL-2-amino-5-phosphonopentanoic acid
BC: Bicuculline
BK: Ca^2+^-activated large conductance K^+^ channel
Ca^2+^: Calcium
CaMKK: Ca^2+^/calmodulin-dependent protein kinase kinase
CTL: Control
DIV: Days *in vitro*
DTX-K: Dendrotoxin-K
h: Hours
HCN: Hyperpolarization activated cyclic nucleotide-gated K^+^ channel
K^+^: Potassium
NMDA: *N*-Methyl-D-aspartate
VGCC: Voltage-gated Ca^2+^ channel
QPCR: Real-time quantitative polymerase chain reaction
SK: Ca^2+^-activated small conductance K^+^ channel
THIK2: Tandem pore domain halothane-inhibited K^+^ channel 2
TRAAK: TWIK-related arachidonic acid-stimulated K^+^ channel
TREK1: TWIK-related K^+^ channel 1
TTX: Tetrodotoxin
XE991: XE991 dihydrochloride
