# Supplementary figures and images for: N-methyl-D-aspartate receptors mediate activity-dependent down-regulation of potassium channel genes during the expression of homeostatic intrinsic plasticity
Source: Mol Brain. 2015 Jan 20;8:4. doi: 10.1186/s13041-015-0094-1 (PMC4333247; doi:10.1186/s13041-015-0094-1)

# Figure S1

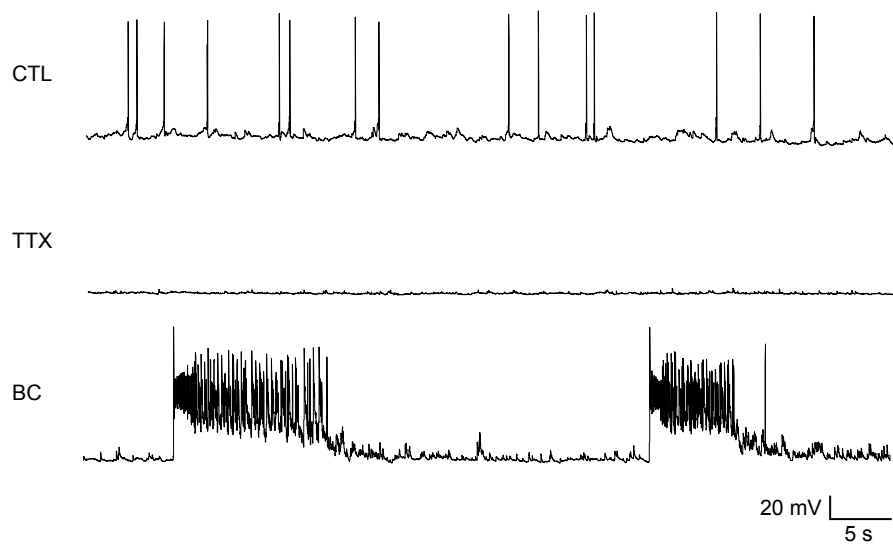

Supplement: Additional file 1: Figure S1. — Effects of acute TTX or BC treatment on spontaneous firing of hippocampal pyramidal neurons. (A) Whole-cell patch clamp recording of rat dissociated hippocampal neurons cultured at high density (DIV 12–14) revealed that acute application of TTX (0.5 μM) rapidly blocks spontaneous firing of AP whereas acute application of BC (20 μM) leads to burst firing compared to CTL-H2O treatment. [file 13041_2015_94_MOESM1_ESM.pdf]

# Figure S2

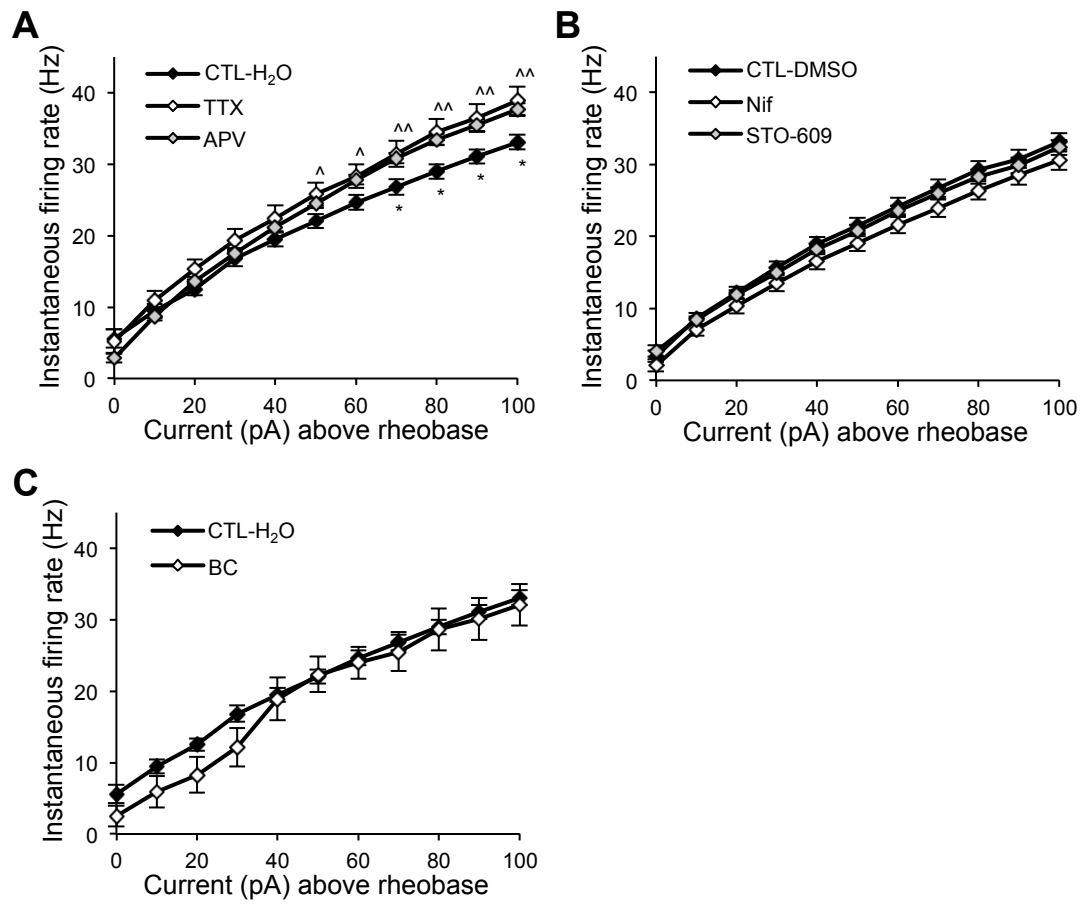

Supplement: Additional file 3: Figure S2. — The AP firing rates in response to current injections from rheobase to rheobase + 100 pA. Average AP firing rates (Hz) shown in Figures 1, 3 and 4 were recalculated in response to current injections from rheobase to rheobase +100 pA. (A) Treatment of hippocampal neurons for 48 h with TTX (n = 10) or APV (n = 9) significantly increased AP firing frequency compared to CTL-H2O treatment (n = 22). (B) Treatment for 48 h with Nif (n = 9) or STO-609 (n = 10) did not alter AP firing frequency compared to CTL-DMSO treatment (n = 12). (C) BC application for 48 h (n =9) did not change AP firing frequency compared to CTL-H2O application (n = 22). Mean ± SEM (*p < 0.05, **p < 0.01 for CTL vs. TTX; ^p < 0.05, ^^p < 0.01 for CTL vs. APV). [file 13041_2015_94_MOESM3_ESM.pdf]

# Figure S3

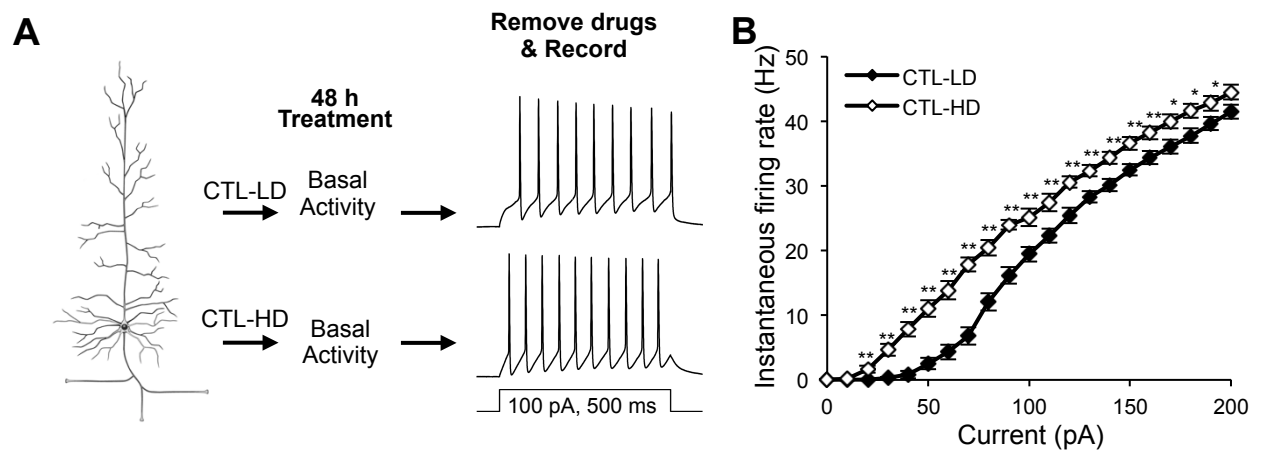

Supplement: Additional file 4: Figure S3. — Effect of culture density on innate excitability of hippocampal pyramidal neurons. (A,B) Whole-cell patch clamp recording of rat dissociated hippocampal neurons cultured at high density and low density (DIV 12–14) after 48 h application of vehicle control (CTL, 0.1% H2O). Following treatment removal, spike trains were evoked in pyramidal neurons by delivering constant somatic current pulses for 500 ms duration at a resting potential of −60 mV. (A) Representative spike trains are shown. (B) Average AP firing rates (Hz) were measured in pyramidal neurons cultured at high density (n = 22) and low density (n = 22). Mean ± SEM (*p < 0.05, **p < 0.01 for CTL-LD vs. CTL-HD). [file 13041_2015_94_MOESM4_ESM.pdf]
