# Supplementary material for: N-methyl-D-aspartate receptors mediate activity-dependent down-regulation of potassium channel genes during the expression of homeostatic intrinsic plasticity
Source: Mol Brain. 2015 Jan 20;8:4. doi: 10.1186/s13041-015-0094-1 (PMC4333247; doi:10.1186/s13041-015-0094-1)
Supplement: Additional file 2: Table S1. — The average rheobase current of hippocampal pyramidal neurons cultured at high-density following 48 h pharmacological treatment. Rheobase (the minimal current that elicited at least one spike) was determined by incremental 10 pA current steps for 500 ms duration at a holding potential of −60 mV. Each value represents the mean ± SEM (**p < 0.01 for CTL-H2O vs. APV). [file 13041_2015_94_MOESM2_ESM.pdf]

Table S1. The average rheobase current of hippocampal pyramidal neurons cultured at high-density following 48 h pharmacological treatment.

Treatment	Rhobase (pA)
CTL-H_2O	33.7 ± 4.1
TTX	26.6 ± 5.6
BC	45.5 ± 7.2
APV	12.7 ± 1.5**
CTL-DMSO	24.4 ± 4.6
Nif	15.1 ± 1.4
STO-609	21.0 ± 2.6

Rheobase (the minimal current that elicited at least one spike) was determined by incremental 10 pA current steps for 500 ms duration at a holding potential of -60 mV. Each value represents the mean ± SEM (***p* < 0.01 for CTL-H_2O vs. APV).
