# Supplementary material for: N-methyl-D-aspartate receptors mediate activity-dependent down-regulation of potassium channel genes during the expression of homeostatic intrinsic plasticity
Source: Mol Brain. 2015 Jan 20;8:4. doi: 10.1186/s13041-015-0094-1 (PMC4333247; doi:10.1186/s13041-015-0094-1)
Supplement: Additional file 5: Table S2. — Passive properties of hippocampal pyramidal neurons cultured at low density. n, number; Cm, Whole-cell membrane capacitance; Rin, input resistance; Vm, resting membrane potential. Each value represents the mean ± SEM. [file 13041_2015_94_MOESM5_ESM.pdf]

Table S2. Passive properties of hippocampal pyramidal neurons cultured at low density.

Treatment	n	C_m (pF)	R_{in} (MΩ)	V_m (mV)
CTL	22	48.2 ± 1.9	485 ± 24	-61.2 ± 0.7

n, number; Cm, Whole-cell membrane capacitance; Rin, input resistance; Vm, resting membrane potential. Each value represents the mean ± SEM.
