# Supplementary material for: N-methyl-D-aspartate receptors mediate activity-dependent down-regulation of potassium channel genes during the expression of homeostatic intrinsic plasticity
Source: Mol Brain. 2015 Jan 20;8:4. doi: 10.1186/s13041-015-0094-1 (PMC4333247; doi:10.1186/s13041-015-0094-1)
Supplement: Additional file 6: Table S3. — AP properties of hippocampal pyramidal neurons cultured at low density. VT, voltage threshold for action potential; AP, Action potential; rise, 10-90% rise time of AP; decay, 10-90% decay time of AP; HW, half-width; fAHP, fast after-hyperpolarization. AP properties were measured from the first action potential evoked by a current step to 100 pA at a holding potential of −60 mV. Each value represents the mean ± SEM (*p < 0.05 for CTL-LD vs. CTL-HD). [file 13041_2015_94_MOESM6_ESM.pdf]

Table S3. AP properties of hippocampal pyramidal neurons cultured at low density.

Treatment	V_T (mV)	AP height (mV)	AP rise (ms)	AP decay (ms)	AP HW (ms)	fAHP (mV)	AP latency (ms)
CTL	-36.9 ± 0.6*	62.8 ± 2.0	0.65 ± 0.03	1.60 ± 0.08	1.79 ± 0.08	-20.2 ± 0.9	47.1 ± 12.6*

V_T, voltage threshold for action potential; AP, Action potential; rise, 10-90% rise time of AP; decay, 10-90% decay time of AP; HW, half-width; fAHP, fast after-hyperpolarization. AP properties were measured from the first action potential evoked by a current step to 100 pA at a holding potential of -60 mV. Each value represents the mean ± SEM (*$p < 0.05$ for CTL-LD vs. CTL-HD).
