# Supplementary material for: N-methyl-D-aspartate receptors mediate activity-dependent down-regulation of potassium channel genes during the expression of homeostatic intrinsic plasticity
Source: Mol Brain. 2015 Jan 20;8:4. doi: 10.1186/s13041-015-0094-1 (PMC4333247; doi:10.1186/s13041-015-0094-1)
Supplement: Additional file 7: Table S4. — Primer pairs for QPCR analysis. Primers were designed with Primer3 software (Whitehead Institute). Primer efficiency was within 90-110% on a standard curve (slope of −3.30 = 100% optimal efficiency), with an R2 correlation coefficients > 0.99, and threshold cycle (CT) values > 8 and < 35. [file 13041_2015_94_MOESM7_ESM.pdf]

Table S4. Primer pairs for QPCR analysis.

Symbol	Description	qPCR Primer Sequence (5')	qPCR Primer Sequence (3')
ARC	activity-regulated cytoskeleton-associated protein	CCAAGAAGTGGTGGGAGTTC	CTGGTACAGGTCCCGCTTAC
BDNF	brain derived neurotrophic factor	GATGAGGACCAGAAGGTTCG	TGTGACCCACTCGCTAATACT
CAMK4	Ca^{2+}/calmodulin-dependent protein kinase IV	CTGGACCTGGAGAGCTATGG	AAGCTGAGTCGAGGGAACTG
CRH	corticotropin releasing hormone	GCCCATCTCTCTGGATCTCAC	TCTCCATCAGTTTCCTGTTGC
GABRD	gamma-aminobutyric acid (GABA) A receptor, delta	TGGACTAATGGAGGGCTACG	GGGTCTCGTTGGTATGGTTG
GAPDH	glyceraldehyde-3-phosphate dehydrogenase	GTCGGTGTGAACGGATTTG	CTTGCCGTGGGTAGAGTCAT
GRIA4	glutamate receptor, ionotrophic, AMPA 4	CCAAGCAGCGTTCAAATAGG	TGGCAGTCTCAATGTTGTCC
GRIN2A	glutamate receptor, ionotropic, NR2A	CCTGGAAGAGGCAGATTGAC	GCAGCCAGCATGTAGAACAC
GRM1	glutamate receptor, metabotropic 1	CGGAGGGATCACAATAAAGC	CAAGAGGTGTCCAGGTAGGC
GRM8	glutamate receptor, metabotropic 8	CAGCAAGCTCTGTGTCCATC	GGCCTCAACACCACTCTCTC
HOMER1	homer homolog 1	TCTTCCAGATCGACCCAAAC	TCAGAGGAGAATCCCAGTCC
HCN1	hyperpolarization-activated, cyclic nucleotide-gated channel 1	CCAGAAGGATGTGAACACTGG	TGCAGGTTGCTGTGAGAGAG
KCNA1	K^+ voltage-gated channel, shaker-related, 1, $K_v1.1$	GCCGCAGCTCCTCTACTATC	GCGGTGCAGTTACCAGTTC
KCNA4	K^+ voltage-gated channel, shaker-related, 4, $K_v1.4$	CTGGAGAACTCAGGGCACAC	GACAAGCAAAGCATCGAACC
KCNAB3	K^+ voltage-gated channel, shaker-related, beta 1, $K_v\beta3$	CTGGGTCACATTTGGCTCTC	TCCAACCTTTGCTCTTGAGG
KCNIP1	K_v channel-interacting protein 1, KCHIP1	TCACCAAGAGGGAGCTACAAG	TGTCGAAGGCATTGAAGAGG
KCNIP3	K_v channel interacting protein 3, calsenilin, KCHIP3	CTTCTTCTTCAATGCCTTCG	CGCAGGATAGGGTAGGTGTG
KCNJ12	K^+ inwardly-rectifying channel, J, 12, Kir2.2	CATCATCTTCTGGGTCATTGC	GCACTCTTCAGTCACGCATC
KCNMB2	K^+ large conductance Ca^{2+}-activated channel, M, beta 2, $BK\beta2$	GCCATCCTGCTTGGACTG	CAGGCAAGGGTACTGAGAGAG
KCNMB4	K^+ large conductance Ca^{2+}-activated channel, M, beta 4, $BK\beta4$	GCATCCTGTCGCTCTTCATC	GGTGCTGGTCGCTGTGTAG
KCNQ3	K^+ voltage-gated channel, KQT-like, 3, $K_v7.3$	CTTCGTGCTGATTGCTTCTG	CGGTGATGAGTTCTTTGCTG
KCNS3	K^+ voltage-gated channel, delayed-rectifier, S, 3, $K_v9.3$	CCAGAAGCAGAAGGACATGG	CATCTGTGGAGTCAGGATCG
KCNN1	K^+ small conductance Ca^{2+}-activated channel, N, 1	CTGATCTCGCTGGAGTTGG	GCAGATAGAGGCGCAGAAAC
LGI1	leucine-rich, glioma inactivated 1	TGGTACAGGGATACTGATGTGG	AGTCGCCAATGAATCTGGTC
LGI2	leucine-rich repeat LGI family, 2	GGTGGTAGCCCAACTCTTTG	CAGCGATGATGAAGAACGTC
LIN7A	lin-7 homolog a	CAGCAATCCGAGAGGTGTATC	TCAGTCTTTGGCAGTTCGAC
NOS1	nitric oxide synthase 1 (neuronal)	CTGCAAAGCCCTAAGTCCAG	GCAGTGTTCCTCTCCTCCAG
RASGRF	ras protein-specific guanine nucleotide-releasing factor 1	ACCAAGAATGGCGTGATTTC	GCACTGGATGATGTCACTGG
SNCB	beta synuclein	CTGGGAGGAGCTGTGTTCTC	TACGCCTCTGGCTCGTATTC
SCN1B	Na^+ channel, voltage-gated, type I, beta, $Na_v1\beta$	GTGGTGTGGAACGGTAGTCG	GATCTCTGACACGATGGATGC
TRPC7	transient receptor potential cation channel, C, 7	GACTTTGCCCAACGAGACC	TCCAGTAGGTTCCACAAGTGC

Primers were designed with Primer3 software (Whitehead Institute). Primer efficiency was within 90-110% on a standard curve (slope of -3.30 = 100% optimal efficiency), with an R2 correlation coefficients > 0.99, and threshold cycle (CT) values > 8 and < 35.
